# Analysis and Prediction of the Earthquake Frequency Sequence in the Anninghe Fault Zone Based on the SARIMA Model

**DOI:** 10.3390/e28050526

**Published:** 2026-05-06

**Authors:** Xiyu Fang, Yuan Xue

**Affiliations:** 1 School of Mathematical Sciences, Chengdu University of Technology, Chengdu 610059, China; 13438720313@163.com; 2 Geomathematics Key Laboratory of Sichuan Province, Chengdu 610059, China

**Keywords:** Anninghe Fault Zone, earthquake frequency, SARIMA model, time series prediction, seasonal periodicity

## Abstract

The Anninghe Fault Zone is an active, deep–large fault in southwestern China, with a history of multiple strong earthquakes. To reveal the temporal patterns of seismicity and improve medium- to short-term earthquake frequency prediction, this study constructs a quarterly seismic frequency sequence (M ≥ 3.0) from May 1972 to September 2025 and applies the SARIMA (seasonal autoregressive integrated moving average) model for modeling and prediction. The hypothesis is that the frequency sequence exhibits modelable seasonality, trends, and nested periodic structures. The ADF test and Ljung–Box test confirm that the sequence is stationary and non-white noise, satisfying the prerequisites for SARIMA modeling. The centered moving average method is used to extract short-term (1 year), medium-term (5 years), and long-term (10 years) periodic components, and corresponding SARIMA models are constructed. Results show that the medium-period model ARIMA(2,0,1) × (1,0,0)_20_ achieves the best prediction accuracy (RMSE = 0.6868, MAE = 0.6143), followed by the short-period model, while the long-period model yields slightly higher errors. All selected models pass residual white noise tests and parameter significance tests, and exhibit good robustness under different training–test splits. The main innovations are: (1) the first systematic application of SARIMA to earthquake frequency prediction in the Anninghe Fault Zone, and (2) a preliminary physical interpretation of multi-scale periodic components (e.g., seasonal loading, strain accumulation fluctuations). This method offers significant application value in regions with sparse seismic networks or limited precursory data, providing a new statistical tool for regional seismic hazard assessment and disaster mitigation planning.

## 1. Introduction

**Figure 1:**
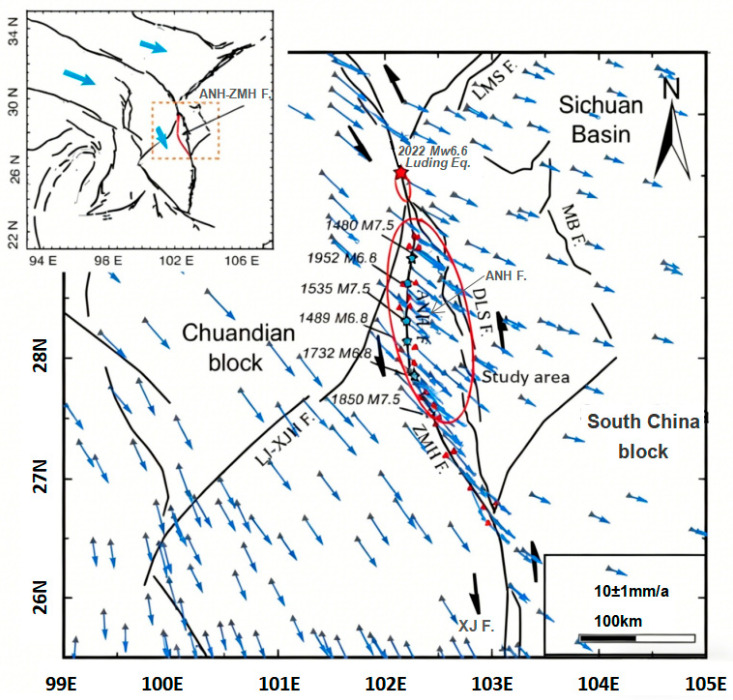
Outline map of the Anning River Fault Zone and its adjacent areas [3]. Black lines indicate active faults obtained from the public data catalog of the China Seismic Experiment Site. Red triangles and gray triangles represent the 24 newly developed near-field GNSS stations and the existing stations used for constraining the kinematic coupling model, respectively. Cyan stars denote historical events (M > 6.5) that occurred in the past 500 years. Blue vectors show the GNSS velocity in this region. Thick black half-arrows indicate the fault kinematic directions. Cyan vectors in the upper-left inset show the block motions. The red star and red ellipse represent the epicenter and rupture extent of the 2022 Mw 6.6 Luding earthquake, respectively. Abbreviations: ANHF: Anninghe Fault; ZMHF: Zemuhe Fault; DLSF: Daliangshan Fault; LJ-XJHF: Lijiang–Xiaojinhe Fault; LMSF: Longmen Shan Fault; MBF: Mabian Fault; XJF: Xiaojiang Fault.

The Anning River Fault Zone is located at the southeastern edge of the Qinghai–Xizang Plateau, running along the border between Sichuan and Yunnan provinces, and is oriented in a north–south direction. It has a total length of approximately 400 kilometers and is a highly active, deep, large fault (Figure 1). This fault forms the eastern boundary of the Sichuan–Yunnan rhombic block and is mainly composed of the Zemu River Fault, the main branch of the Anning River Fault, and the Daliangshan Fault, among other secondary faults. It has both reverse thrust and right-lateral strike-slip properties. Historically, this fault zone has experienced frequent strong earthquakes, including the 1536 Xichang North 7.5 magnitude earthquake and the 1850 Xichang 7.5 magnitude earthquake. In recent years, there have been continuous minor and medium earthquakes, indicating a relatively high seismic hazard. Along the fault zone, there are important towns such as Xichang, Mengniang, and Dechang. These towns are close to population centers such as Panzhihua and Liangshan Prefecture. Previous studies have shown that the frequency and intensity of earthquakes in this area are higher than those in the adjacent Daliangshan Fault Zone, and the probability of strong earthquakes in sections such as Puge and Mianning is relatively high [1]. Zhou et al. [2] also provided a basis for earthquake tectonic environment evaluation from the perspectives of fault zone characteristics, historical earthquakes, and focal displacement. However, research on time series modeling and prediction of the seismicity frequency of this fault zone is still relatively limited. Therefore, a systematic analysis of its frequency characteristics and temporal patterns, and an assessment of future earthquake probabilities, have important scientific value and practical significance for seismic prevention planning along the line.

The formation of earthquakes is closely related to plate motion. According to the elastic rebound theory [4], when mantle material undergoes thermal convection, crustal plates squeeze against each other, leading to continuous accumulation of crustal stress. When the stress exceeds the strength limit of the fault zone, the crust suddenly ruptures and releases energy, resulting in an earthquake. Thereafter, the plates continue to move, and stress accumulates again, repeating the cycle. This complete process is referred to as the seismic cycle, which generally denotes the entire process of stress accumulation, slip release, and re-accumulation on the same fault zone from one major earthquake to the next [5]. On a given fault zone, seismic activity may exhibit alternating periods of high activity and quiescence in terms of frequency. This regular time interval is known as the characteristic earthquake recurrence interval, representing the average time between characteristic earthquakes on that fault zone [6]. For the study area, existing research [7] supports the above earthquake preparation mechanism, indicating that plate compression and stress accumulation are the main drivers of local seismic activity, and that seismic activity on this fault zone indeed exhibits periodic alternation between active and quiescent phases.

Due to the complexity of earthquake formation mechanisms, it is currently impossible to achieve precise earthquake prediction. However, analyzing historical earthquake data and studying the frequency distribution and periodic characteristics of earthquake events on the time axis can provide important clues for revealing patterns of earthquake activity. Previous studies have shown that seismic activity is not completely random in time, but exhibits certain periodic or rhythmic characteristics [8,9]. Exploring the periodic patterns of earthquake frequency can help deepen the understanding of regional earthquake activity trends and provide a scientific basis for disaster prevention and mitigation.

This paper studies the seismicity frequency pattern of the Anning River Fault Zone, which has clear temporal attributes. Previous statistical models applied to earthquake catalog data—such as Woessner and Wiemeron [10] on completeness, Griffin et al. [11] on periodicity and clustering, Hainzl et al. [12] and Touati et al. [13] on interevent-time distributions, and Zhuang et al. [14] on temporal seismicity models—often assume linearity or stationarity, limiting their ability to capture the complex nature of earthquake occurrence. The seasonal autoregressive integrated moving average (SARIMA) model, an extension of the autoregressive integrated moving average (ARIMA) model, has notable shortcomings for predicting earthquake timing: it assumes fixed seasonality rarely seen in seismicity, struggles with clustered and aftershock-dominated sequences, and fails to capture the long-memory dependence highlighted by Hainzl et al. [12] and Touati et al. [13]. Therefore, acknowledging these limitations, this paper applies SARIMA to recent earthquake catalog data from the Anning River Fault Zone to explore and predict its seismicity frequency pattern.

## 2. Relevant Theories and Methods

### 2.1. G-R Relationship Equation

In the assessment of earthquake hazard, seismic activity parameters play an indispensable role. In the field of statistical seismology, the magnitude–frequency relationship (Gutenberg–Richter relation, G-R) proposed by Gutenberg and Richter is one of the most fundamental laws [15]:

(1) lgN=a−bM

where *M* represents the magnitude of the earthquake; *N* represents the cumulative number of seismic events greater than the magnitude *M*; *a* is a constant; and the slope value *b* represents the proportion of seismic frequencies at each magnitude relative to the total seismic frequency, and it is an important parameter for earthquake activity. A low value of *b* indicates that earthquakes of higher magnitudes account for a large proportion among all earthquakes, while a high value of *b* indicates that small earthquakes account for a larger proportion.

Since Gutenberg et al. proposed Formula (1) and applied it to global seismic activity, this relationship has been widely adopted worldwide. It is the most frequently cited empirical relationship in seismological studies and has been extensively applied in earthquake activity analysis, geological research, and earthquake hazard prediction [16].

### 2.2. SARIMA Model

The ARIMA model was first proposed by Box and Jenkins in the 1970s. Its full name is the autoregressive integrated moving average model, denoted as ARIMA(p,d,q). Here, p and q represent the autoregressive and moving average orders respectively; d represents the trend difference order. The basic model structure is as follows:

(2) $$\Phi \left(B\right)=\nabla^{d}x_{t}=\Theta \left(B\right)\varepsilon_{t}$$

where $\Phi \left(B\right)=1-\Phi_{1}B-\cdots -\Phi_{p}B^{p}\nabla^{d}={\left(1-B\right)}^{d}$, p are polynomials of autoregressive coefficients of order *p*; B is the lag operator, with $Bx_{t}=x_{t-1}$; $\nabla^{d}$ is the differencing of order *d*; and $\Theta \left(B\right)=1-\theta_{1}B-\cdots -\theta_{q}B^{q}$ is the polynomial of moving average coefficients of order *q* [17].

The ARIMA model can model non-stationary time series without seasonal effects. However, many time series in real life have certain periodicity. For sequences that contain both seasonal effects and long-term trend effects with complex interactive relationships between them, the SARIMA model can be adopted. The SARIMA model, also known as the multiplicative seasonal model (ARIMA multiplicative model), was first proposed with a general expression by Wang et al. [18], denoted as ARIMA(p,d,q) × (P,D,Q)_s_, where P and Q are the orders of seasonal autoregression and seasonal moving average, respectively; D is the order of seasonal differencing; and s is the seasonal period. As an extension of the ARIMA model, this model performs seasonal differencing on the sequence based on the original model to extract seasonal information, so as to achieve the purpose of modeling seasonal time series.

For the time series $x_{t}$, the expression of the SARIMA model can be denoted as:

(3) $$\nabla^{d}\nabla_{s}^{D}x_{t}=\frac{\theta \left(B\right)\theta_{s}\left(B\right)}{\Phi \left(B\right)\Phi_{s}\left(B\right)}\varepsilon_{t}$$

where $\nabla_{s}^{D}$ is the D-th order seasonal difference with s as the period; $\varepsilon_{t}$ is the random interference of the error term t at the moment; and $\Phi_{s}\left(B\right)$ and $\theta_{s}\left(B\right)$ represent the polynomial of the P-th order seasonal autoregressive coefficient and the polynomial of the Q-th order seasonal moving average coefficient, respectively.

## 3. Research Methods and Modeling Process

**Figure 2:**
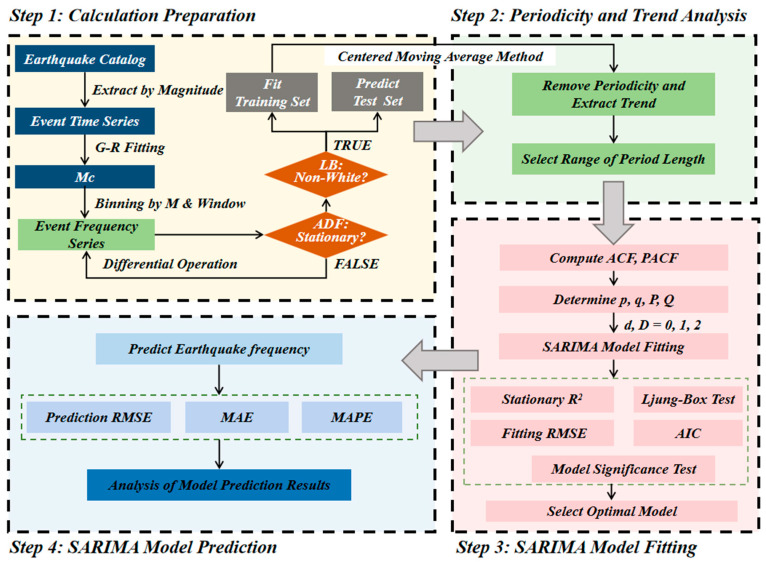
Analysis and prediction process of the SARIMA model for earthquake frequency.

To obtain the periodicity, trend, and other temporal patterns of earthquake occurrence frequencies (defined as the number of earthquakes within different time windows: annual, quarterly, and monthly), and to predict the frequency of earthquakes within a certain period, this paper constructs an analysis and prediction method based on the SARIMA model. The overall process is shown in Figure 2.

Firstly, the earthquake occurrence frequency sequence (based on the above-defined annual, quarterly, and monthly time windows) is constructed based on the earthquake catalog data, and a stationary non-white noise sequence is selected through the ADF test and LB test. It is proportionally divided into a training set and a validation set. On this basis, different-order centered moving average methods are used to remove the periodicity and extract the trend of the training set sequence, to identify short-, medium-, and long-cycle characteristics, and thereby determine the type of the SARIMA model (additive or multiplicative). Further, by analyzing the autocorrelation coefficient (ACF) and partial autocorrelation coefficient (PACF) of the sequence, the non-seasonal and seasonal parameter orders (*p*, *q*, *P*, *Q*) of the model are preliminarily determined, and the candidate model set is constructed by combining the difference order (*d*, *D*). The candidate models are fitted, and residual white noise tests are conducted to select the model that passes the test and has the largest stationary R^2^ as the optimal model. Finally, the optimal model is used to predict the validation set, and the prediction effect is evaluated by indicators such as the Root Mean Square Error (RMSE), thereby achieving the prediction of future earthquake frequencies.

## 4. Overview of the Study Area and Data Preprocessing

This article collects and organizes catalog data of earthquakes with magnitudes of 2.5 and above that occurred within the longitude and latitude range of the Anning River Fault Zone (26.96–29.06° N, 102.14–102.91° E) from 7 May 1972 to 30 September 2025, totaling 291 entries from the China Earthquake Networks Center (https://www.cenc.ac.cn/) (last accessed on 12 March 2026).

Based on the characteristics and practical application results of existing completeness analysis methods, this study first employs the maximum likelihood method (MLE) [19] to fit the Gutenberg–Richter (G-R) relationship and estimate the b-value (i.e., the slope of the G-R curve) for different magnitude thresholds M_cut_. To quantitatively determine the minimum magnitude of completeness M_C_, the Root Mean Square Error (RMSE) between the observed and predicted cumulative frequencies (in logarithmic scale) is calculated for each M_cut_. The RMSE is defined as:

(4) $$RMSE=\sqrt{\frac{1}{k}\sum_{i=1}^{k}{\left(\mathrm{lg}N_{obs,i}-\mathrm{lg}N_{pred,i}\right)}^{2}}$$

where k is the number of magnitude bins with M ≥ M_cut_. The value of M_cut_ that minimizes the RMSE is taken as M_C_.

**Figure 3:**
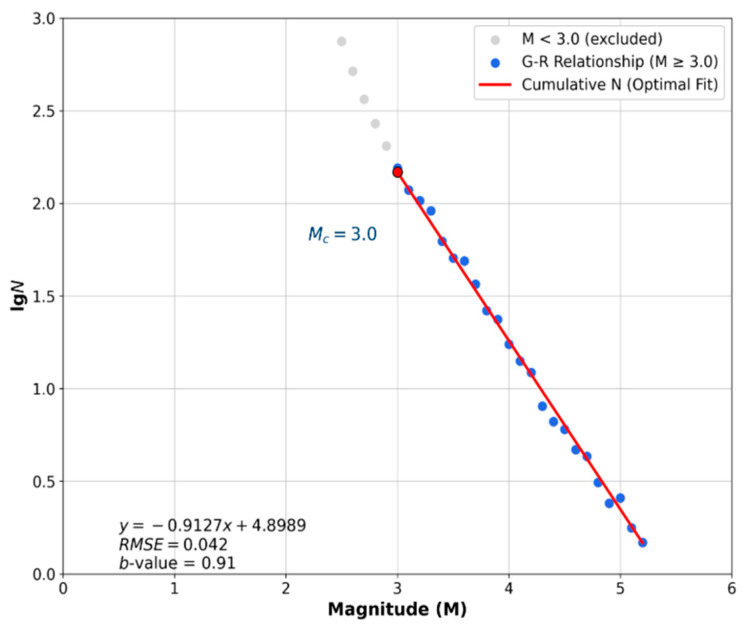
Fitting effect of the G-R relationship for the earthquake frequency sequence.

Table 1 reports the b-values (estimated via MLE) and the corresponding RMSE for M_cut_ ranging from 2.5 to 3.4. The RMSE reaches its minimum of 0.042 at M_cut_ = 3.0, and the b-value stabilizes around 0.91 for M ≥ 3.0. For M_cut_ < 3.0, the RMSE is consistently higher (≥0.071), indicating a poorer G-R fit due to incomplete detection of smaller events. Therefore, the minimum magnitude of completeness of this catalog is determined as M_C_ = 3.0. Figure 3 shows the G-R fitting curve for M ≥ 3.0 as a visual reference, with an RMSE of 0.042.

**Figure 4:**
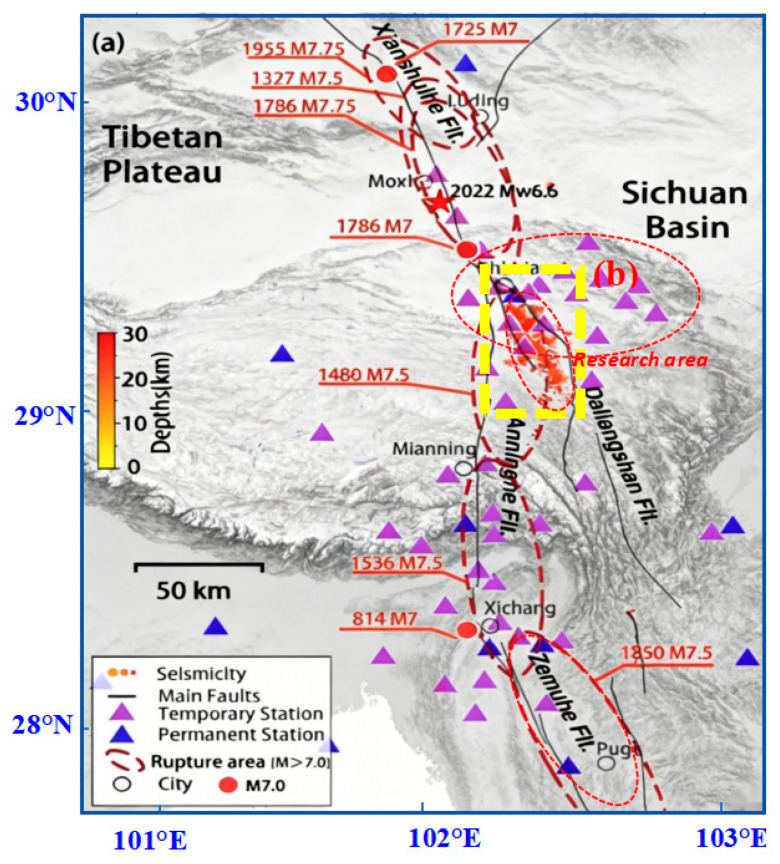
Distribution of earthquakes and stations in the Anning River Fault Zone and its adjacent areas [20]. (**a**) represents the overall topographic environment and characteristics of the entire region, and (**b**) denotes the core study area selected in this research (Yellow dashed box). The red star represent the epicenter extent of the 2022 Mw 6.6 Luding earthquake.

A total of 139 earthquake catalog data were obtained for the analysis. The time range of the earthquakes was from 7 May 1972, to 30 September 2025, and the magnitude range was from 3.0 to 5.2. The data grouped by magnitude are shown in Table 2, and the spatial distribution of the earthquake locations is depicted in Figure 4.

According to the current classification of earthquake magnitudes and intensities by scholars, earthquakes with magnitudes less than 3.0 are classified as weak earthquakes, which are generally not easily detectable by people; earthquakes with magnitudes greater than 3.0 but less than or equal to 4.5 are considered perceptible earthquakes; earthquakes with magnitudes greater than 4.5 but less than or equal to 6.0 are classified as moderately weak earthquakes, which can cause damage; earthquakes with magnitudes greater than 6.0 are classified as strong earthquakes. Based on the above criteria, combined with the characteristics of the sample data (there are fewer high-magnitude events in the data, making it difficult to conduct research), and considering that the M_C_ obtained from the previous text is 3.0, the modeling of M ≥ 2.5 (all data) will not be carried out in subsequent practical work, and the magnitude groups of the data are determined as M ≥ 3.0, M ≥ 4.0, and M ≥ 5.0 (Table 3).

First, the three magnitude groups M ≥ 3.0, M ≥ 4.0, and M ≥ 5.0 are abbreviated as M3, M4, and M5, respectively. Then, the statistical and sorting work is carried out on an annual, quarterly, and monthly basis. In Table 4, the time periods of the quarterly and monthly sequences are named “Annual”, “Quarter” and “Monthly”. In Table 5, the ADF test indicates that all sequences are stationary and no differencing is required. Subsequently, the LB test shows that the quarterly sequence M3, the monthly sequence M3, and the monthly sequence M4 are non-white noise sequences, while the remaining sequences are white noise sequences. Due to the excessive zero values in the monthly sequences M3 and M4, and since the data in the test set are all zero, modeling and prediction cannot be carried out. Therefore, only the quarterly sequence M3 is analyzed.

## 5. Model Establishment and Parameter Optimization

**Figure 5:**
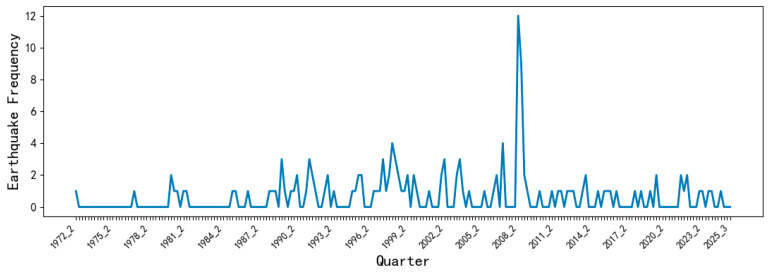
Quarterly M_3_ seismic frequency sequence diagram.

**Figure 6:**
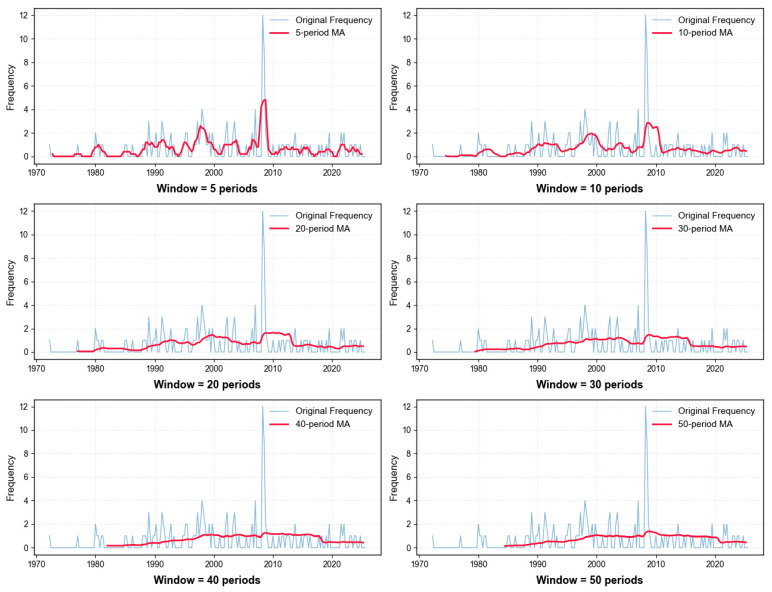
Results of seismic frequency sequence trend extraction using the central moving average method.

As shown in Figure 5, the quarterly seismic occurrence frequency sequence M_3_ generally shows a trend of first increasing and then decreasing, and there are changes in short-, medium- and long-nested cycles. Additionally, the seismic occurrence frequency suddenly increased at the time point 2008_2. These earthquakes were actually the main shock and aftershocks of the magnitude 8.0 Wenchuan earthquake on 12 May 2008. To fit the optimal SARIMA model, it is necessary to analyze and determine the specific periods of the sequence’s cycle changes and further confirm its overall trend. An attempt was made to use the 5th-, 10th-, 20th-, 30th-, 40th-, and 50th-order (period) centered moving average methods [17] to remove cycles and extract the trend from the sequence. The results are shown in Figure 6.

As shown in Figure 6, the frequency of earthquakes of magnitude 3.0 and above along the Anning River Fault Zone fluctuated at a low level from 1972 to 2000. It gradually increased after 2000, reached its peak in 2008, then declined and remained at a lower level. Therefore, this paper adopts the multiplicative SARIMA model [21]. Additionally, the 5th-, 20th-, and 40th-order centered moving average methods were used to remove short-, medium-, and long-term periodicities and extract the trend, which achieved better results. The 50th-order centered moving average method was the best for removing long-term periodicities and extracting the trend, but the period number was too long and was not suitable for calculation, nor was it necessary. Therefore, the period numbers of 4–6, 19–21, and 39–41 were selected for SARIMA model fitting to determine the short-, medium-, and long-term models with optimal parameters. The training set data used for fitting was the first 90% (time period from 1972_2 to 2020_2). 

**Figure 7:**
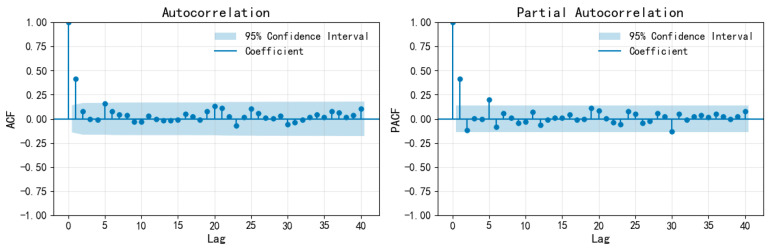
ACF and PACF plots of the M_3_ training set for the quarterly earthquake frequency sequence.

**Figure 8:**
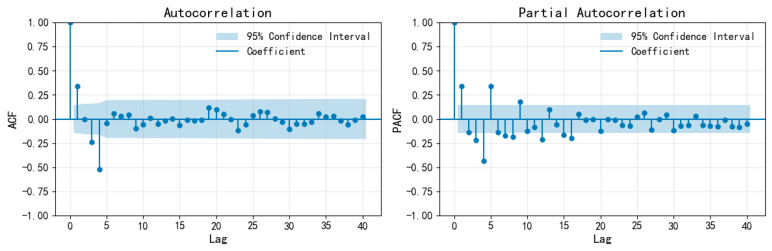
ACF and PACF plots of the M_3_ training set for the quarterly earthquake frequency sequence after fourth-order seasonal differencing.

First, consider that the seasonal cycle is a short period. Since the data are quarterly, let us take s = 4. The autocorrelation function (ACF) and partial autocorrelation function (PACF) plots of the training set of the quarterly seismic frequency sequence M_3_ are shown in Figure 7, and those after fourth-order seasonal differencing are shown in Figure 8. Based on the autocorrelation and partial autocorrelation coefficients of the training set of the quarterly seismic occurrence frequency sequence M_3_ and its seasonally differenced sequence, the model order is determined as ARIMA(4,d,0) × (0,0,1)_4_, where d is the difference order. Taking d = 0, 1, and 2 for model fitting, the model fitting and parameter test (Table 6) show that only ARIMA(4,1,0) × (0,0,1)_4_ passes the parameter test and residual white noise test (significance > 0.05), and has the highest stationary R_2_. Therefore, it is determined that d = 1.

Furthermore, the number of short cycles is set to 5 and 6, the number of medium cycles to 19, 20, and 21, and the number of long cycles to 39, 40, and 41. Based on the autocorrelation coefficient and partial autocorrelation coefficient of the sequence, each SARIMA model is ordered and fitted. The model fitting parameters are shown in Table 7. The *p*-values of the LB test for each cycle model are all greater than 0.05, indicating that the residual white noise test is passed and that the models are significant. By further comparing the stationary R-squared values of the short-term, medium-term, and long-term cycle models, and selecting the models that pass the parameter test and have the largest stationary R-squared values, the optimal models for the short-term, medium-term, and long-term cycles are obtained as ARIMA(2,0,1) × (1,1,0)_4_, ARIMA(2,0,1) × (1,0,0)_20_, and ARIMA(5,1,2) × (1,1,1)_40_, respectively.

Table 7 shows that the standard deviations of stationary R^2^, AIC, and fitting RMSE within the neighborhoods of each cycle type are all small. Among them, the maximum standard deviation of AIC is 7.2 (short cycle), the maximum standard deviation of fitting RMSE is 0.0084 (long cycle), and the maximum standard deviation of stationary R^2^ is 0.0037 (long cycle). Overall, the model performance remains stable under small variations in the cycle parameter, indicating that the selected SARIMA models are insensitive to cycle length and possess good local stability.

## 6. Prediction Results and Analysis

**Figure 9:**
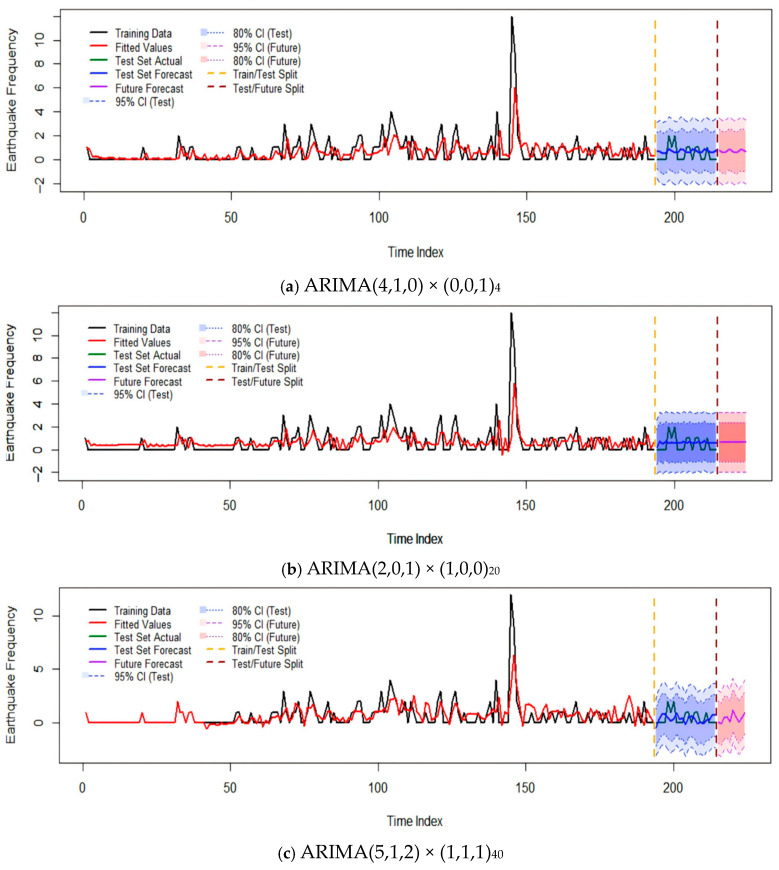
Fitting effects of different cycle models.

The SARIMA models of different cycles obtained from the previous fitting were used to predict the last 10% of the seismic frequency (seismic order: 194–214) of the quarterly seismic frequency sequence M_3_, while the first 90% of the seismic frequency was used for ARIMA model fitting. The fitting effects of the models for different cycles of the sequence is shown in Figure 9, and the Root Mean Square Error (RMSE) predicted by each model is shown in Table 8.

**Figure 10:**
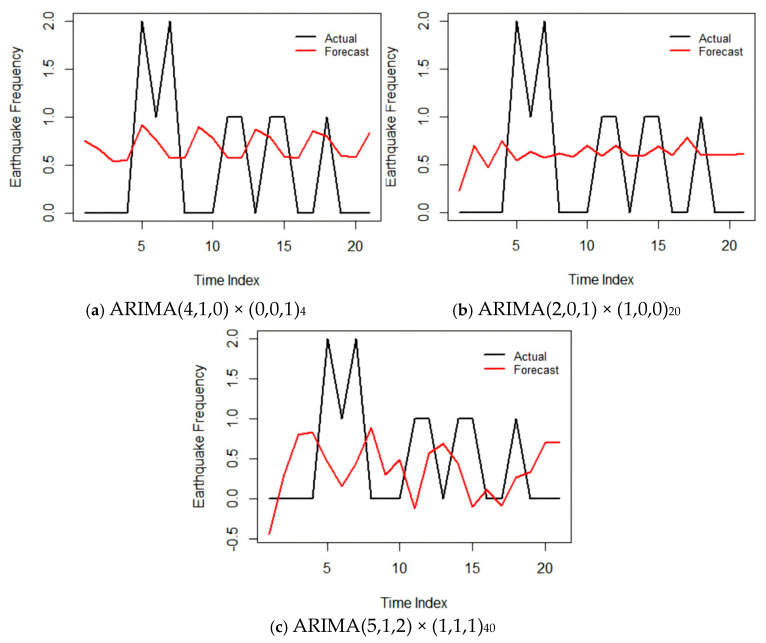
Prediction effects of models with different cycles.

The optimal model was used to predict the test set data of each sequence, and the predicted results were compared with the actual values, as shown in Figure 10.

To examine the dependence of model performance on the partitioning of training and test sets, three different fixed split schemes (training proportions of 90%, 80%, and 70%) were adopted. The prediction RMSEs of the three models on the corresponding test sets are compared, and the results are shown in Table 9.

Table 9 shows that the prediction RMSE of all models increases to varying extents as the training proportion decreases (i.e., as the test set becomes larger). Specifically, the RMSE of the short-cycle model increases from 0.7045 to 0.7412 (an increase of approximately 5.2%), with a standard deviation of 0.0154; the RMSE of the medium-cycle model increases from 0.6868 to 0.7124 (an increase of approximately 3.7%), with a standard deviation of 0.0106; and the RMSE of the long-cycle model increases from 0.7983 to 0.8631 (an increase of approximately 8.1%), with a standard deviation of 0.026. Among the three models, the medium-cycle model exhibits the smallest fluctuation and the lowest standard deviation (0.0106) across different split schemes, indicating that its predictive performance is relatively insensitive to the choice of training–test split and thus possesses good robustness. The long-cycle model shows the largest standard deviation (0.026), reflecting higher sensitivity to the split point, and its prediction results should be interpreted with caution. The sensitivity of the short-cycle model lies between the two.

The detailed 10-step prediction results for the short-term, medium-term, and long-term models are presented in Table 10, Table 11 and Table 12, respectively.

Figure 10 shows the comparison between the predicted values and actual values of the three models on the test set. Overall, all models can well capture the overall variation trend of seismic frequency, but there are differences in prediction accuracy in specific quarters, which are manifested as follows: (1) The predicted values of the short-cycle model ARIMA(4,1,0) × (0,0,1)_4_ fluctuate frequently, which can reflect short-term changes in seismic activity to a certain extent, but there are large deviations in individual periods such as the third quarter of 2021 and the first quarter of 2023. The RMSE of this model is 0.7045, the MAE is 0.6420, and the MAPE is 39.53%, indicating that short-term prediction is acceptable in most quarters, but its responsiveness to sudden seismic activities (such as aftershock sequences) is limited.

(2) The prediction curve of the medium-cycle model ARIMA(2,0,1) × (1,0,0)_20_ is relatively smooth, with a high degree of agreement with the actual value sequence. Especially during 2020–2022, the predicted values basically fall near the actual frequency. The RMSE of this model is 0.6868, the MAE is 0.6143, and the MAPE is 45.45%, which has the smallest error among the three and shows the best prediction performance. This indicates that the seasonal law of the medium cycle (about five years) is relatively stable in the seismic activity of the Anninghe Fault Zone, and the model can effectively extract the periodic characteristics at this scale.

(3) The long-cycle model ARIMA(5,1,2) × (1,1,1)_40_ has a relatively large prediction error, with an RMSE of 0.7983, an MAE of 0.6941, and an MAPE of 47.81%. The prediction curve deviates significantly from the actual values in the later period of the test set (2024–2025), which may be related to the sparse distribution of seismic events and insufficient sample size within the long-cycle seasonal window (40 quarters, i.e., 10 years). In addition, the long-cycle model is more sensitive to trend changes. As seismic activity has tended to be calm in recent years, the model may have over-extrapolated historical trends.

In summary, the SARIMA model shows certain effectiveness in predicting the seismic frequency of the Anninghe Fault Zone, but the prediction error is still affected by the following factors: (1) Data sparsity: Earthquakes with M ≥ 3.0 are mostly 0 or 1 time on a quarterly scale, with a high proportion of zero values, leading to large sequence volatility and making it difficult for the model to fit accurately. (2) External interference events: The aftershock activity triggered by the 2008 Wenchuan Earthquake formed a significant peak in the sequence, and such sudden events are difficult to be accurately predicted by pure statistical models.

Despite the above limitations, this study still verifies the feasibility of the SARIMA model in seismic frequency prediction and provides a basis for the subsequent introduction of more seismic physical parameters or hybrid models.

## 7. Discussion

Previous studies have shown that earthquake prediction methods based on geophysical precursor anomalies (e.g., ionospheric, electromagnetic, and thermal anomalies) [22,23,24,25] can predict the timing of earthquake occurrence to a certain extent by analyzing anomalous variation characteristics before historical earthquakes, and have achieved high accuracy in specific cases. However, such methods typically require continuous, real-time monitoring of relevant geophysical parameters, demand a high quantity and quality of basic data, and have anomaly discrimination criteria have limited transferability across different fault systems or tectonic settings, making them difficult to apply directly to the analysis of large-scale tectonic seismicity. In view of these limitations, the present study attempts to work from a different perspective, namely, without relying on precursory observations, starting directly from the earthquake catalog itself to analyze the intrinsic statistical structure of the seismic frequency sequence. As a complementary exploration, this method can provide a baseline for seismic frequency prediction independent of observational infrastructure in regions where geophysical precursor data are sparse or unavailable.

Unlike previous ARIMA studies that have mostly focused on magnitude prediction [26] or earthquake occurrence time prediction [27], this study takes the less-attended seismic frequency as its prediction target. The results show that the SARIMA model achieves certain effectiveness in predicting seismic frequency at different periodic scales in the Anninghe Fault Zone. Among them, the medium-period model performs best (RMSE = 0.6868, MAE = 0.6143), the mean absolute percentage error of the short-period model is controlled at 39.53%, and the long-period model exhibits a significantly increased prediction error due to the excessively long seasonal period (40 quarters). This indicates the feasibility of the SARIMA model in frequency prediction and extends its application beyond magnitude and occurrence time prediction.

Within the above data-driven framework, we further attempt to provide a preliminary physical interpretation of the multi-scale periodic structure identified from the sequence, although the following explanations are speculative. Considering that the quarterly M ≥ 3.0 seismic frequency sequence is dominated by small-to-moderate earthquakes (M3.0–3.9, accounting for >80% of the total; see Table 1), these periodicities may reflect seismic clustering behavior at different temporal scales. Among them, the short-term cycle (4–6 quarters, i.e., 1–1.5 years) may be related to modulation of the shallow stress field by seasonal surface loading variations (e.g., rainfall, snow cover, or reservoir impoundment), or may reflect the decay duration of aftershock sequences following moderate earthquakes. The medium-term cycle (20 quarters, i.e., 5 years) is the focus of our attention: we hypothesize that it may be linked to fluctuations in the local strain accumulation rate or episodic slow-slip events on the Anninghe Fault. Previous geodetic studies have shown that this fault is not uniformly locked but exhibits spatially variable slip rates [3,20]; this five-year cycle may represent the characteristic time for stress transfer and triggering among small fault segments or asperities. The long-term cycle (approximately 40 quarters, i.e., 10 years) may correspond to the characteristic recurrence interval of larger earthquakes (M ≥ 5) on the Anninghe Fault Zone, or be associated with longer-term fluctuations in regional tectonic stress loading. It should be emphasized that the above interpretations are speculative and require validation through joint analysis with continuous geodetic time series (e.g., GNSS or InSAR).

Future work may further integrate physical constraints (e.g., b-value or strain rate) into the SARIMA framework to enhance its interpretability and prediction robustness.

## 8. Conclusions

Based on the earthquake catalog data with M ≥ 3.0 in the Anninghe Fault Zone from May 1972 to September 2025, this study constructed a quarterly seismic frequency sequence and used the SARIMA model to model and predict its temporal structure. The main conclusions are as follows:

(1) The quarterly seismic frequency sequence of earthquakes with M ≥ 3.0 in the Anninghe Fault Zone has significant periodic and trend characteristics, which meets the modeling premise of the SARIMA model. The ADF test shows that the sequence is stationary, and the Ljung–Box test rejects the white noise hypothesis, indicating that the sequence has a modelable time-dependent structure.

(2) The nested short-term, medium-term, and long-term periodic characteristics of the sequence were identified by the central moving average method, and corresponding scale SARIMA models were constructed accordingly. The model parameters passed the residual white noise test and parameter significance test, indicating that the model structure is reasonable.

(3) The prediction results show that the medium-cycle model ARIMA(2,0,1) × (1,0,0)_20_ has the best prediction accuracy (RMSE = 0.6868, MAE = 0.6143), followed by the short-cycle model, while the long-cycle model has a slightly higher error. This indicates that the SARIMA model has certain practical value in short-term and medium-term seismic frequency prediction, but there are still limitations in dealing with extreme events and long-cycle fluctuations.

(4) This study verifies the feasibility of the SARIMA model in seismic frequency prediction, expands its application direction beyond magnitude and occurrence time prediction, and provides a new methodological reference for regional seismic activity analysis. In the future, more physical parameters can be integrated, or hybrid models can be introduced to further improve the prediction accuracy and robustness.

## Figures and Tables

**Table 1:** G-R fitting parameters estimated by the maximum likelihood method and RMSE for different magnitude thresholds M_cut_.

M_cut_	b-Value	RMSE	M_cut_	b-Value	RMSE
2.5	0.87	0.151	3.0	0.91	0.042
2.6	0.88	0.133	3.1	0.90	0.058
2.7	0.89	0.115	3.2	0.89	0.074
2.8	0.90	0.097	3.3	0.88	0.092
2.9	0.90	0.071	3.4	0.87	0.111

**Table 2:** Grouped data volume of earthquakes of magnitude 3.0 and above in the Anning River Fault Zone from May 1972 to September 2025.

Group Sequence Number	Magnitude Range (Scale)	Data Volume (Units)
1	3.0–3.4	81
2	3.5–3.9	32
3	4.0–4.4	18
4	4.5–4.9	7
5	≥5.0	1

(Note: Magnitude ranges are closed intervals (e.g., 3.0–3.4 includes M3.0, M3.1, M3.2, M3.3, and M3.4). Data source: China Earthquake Networks Center.).

**Table 3:** Cumulative data volume of each magnitude group.

Group Sequence Number	Magnitude Range	Cumulative Data Volume
1	M ≥ 3	139
2	M ≥ 4	26
3	M ≥ 5	1

**Table 4:** Frequency data of different magnitude groups by different statistical calibers (earthquake events only).

Statistical Scope	Period	M_3_	M_4_	M_5_
Annual	1972	1	1	0
	1977	1	1	0
	…	…	…	…
	2024	2	0	0
Quarter	1972_2	1	1	0
	1977_1	1	1	0
	…	…	…	…
	2024_4	1	0	0
Monthly	1972_05	1	1	0
	1977_01	1	1	0
	…	…	…	…
	2024_11	1	0	0

**Table 5:** Pre-test information of models for each seismic frequency sequence.

Statistical Scope	Magnitude Group	ADF Statistic	ADF Test *p*-Value	Stationary (Yes/No)	LB Statistic	LB Test *p*-Value	White Noise (Yes/No)
Annual	M_3_	−6.069	1.16 × 10^−7^	Yes	3.733	0.589	Yes
Annual	M_4_	−5.698	7.81 × 10^−7^	Yes	3.138	0.371	Yes
Annual	M_5_	−7.280	1.51 × 10^−10^	Yes	0.118	0.999	Yes
Quarter	M_3_	−4.939	2.93 × 10^−5^	Yes	45.681	1.64 × 10^−6^	No
Quarter	M_4_	−12.514	2.64 × 10^−23^	Yes	16.226	0.093	Yes
Quarter	M_5_	−14.595	4.26 × 10^−27^	Yes	0.0514	1.000	Yes
Monthly	M_3_	−10.418	1.73 × 10^−18^	Yes	142.146	2.21 × 10^−24^	No
Monthly	M_4_	−12.729	9.45 × 10^−24^	Yes	22.200	0.035	No
Monthly	M_5_	−25.298	0	Yes	0.019	1	Yes

(Note: ADF test null hypothesis: time series has a unit root (non-stationary). LB test (Ljung–Box) null hypothesis: residuals are white noise. Significance level α = 0.05. “Yes” indicates rejection of null hypothesis (stationary or non-white noise) unless *p*-value > 0.05.

**Table 6:** Fitting parameters of the ARIMA(4,d,0) × (0,0,1)_4_ model.

Model	Stationary R^2^	LB Test *p*-Value	Parameter Test	Fitted RMSE	AIC
ARIMA(4,0,0) × (0,0,1)_4_	0.3041	0.7463	Not Significant	1.1986	615.29
ARIMA(4,1,0) × (0,0,1)_4_	0.3124	0.5333	pass	1.1820	597.8
ARIMA(4,2,0) × (0,0,1)_4_	0.2816	0.0004	pass	1.3569	617.73

(Note: “parameter test” indicates whether all model coefficients are statistically significant (*p* < 0.05). RMSE: Root Mean Square Error (smaller values indicate better model fit). AIC: Akaike Information Criterion (lower value indicates a better fit). LB test *p*-value > 0.05 indicates residuals are white noise, satisfying model assumption.)

**Table 7:** Model parameters and periodic neighborhood stability of the M_3_ SARIMA model for the Anninghe Fault Zone quarterly earthquake frequency.

Cycle	Model	Stationary R^2^	LB Test *p*-Value	Parameter Test	Fitted RMSE	AIC
Short Cycle	ARIMA(4,1,0) × (0,0,1)_4_	0.3124	0.5333	pass	1.1820	597.8
	ARIMA(0,1,2) × (0,0,1)_5_	0.3103	0.9154	pass	1.1840	611.8
	ARIMA(0,1,2) × (0,0,1)_6_	0.3098	0.9103	pass	1.1826	612.5
Short-Cycle Mean ± SD	-	−0.3108 ± 0.0014	-	-	1.1829 ± 0.0009	607.4 ± 7.2
Medium Cycle	ARIMA(2,0,1) × (1,0,0)_19_	0.3010	0.6754	pass	1.2257	583.3
	ARIMA(2,0,1) × (1,0,0)_20_	0.3075	0.4375	pass	1.2249	582.1
	ARIMA(2,0,1) × (1,0,0)_21_	0.3034	0.4320	pass	1.2253	584.2
Medium-Cycle Mean ± SD	-	0.3040 ± 0.0027	-	-	1.2253 ± 0.0003	583.2 ± 0.9
Long Cycle	ARIMA(5,1,2) × (1,1,1)_39_	0.2573	0.6764	pass	1.3325	617.9
	ARIMA(5,1,2) × (1,1,1)_40_	0.2579	0.6859	pass	1.3125	617.7
	ARIMA(5,1,2) × (1,1,1)_41_	0.2498	0.6923	pass	1.3276	618.4
Long-Cycle Mean ± SD	-	0.2550 ± 0.0037	-	-	1.3242 ± 0.0084	618.0 ± 0.3

(Note: SD = standard deviation. Stationary R^2^ measures the proportion of variance explained by the model on stationary-transformed data. Models with LB test *p*-value > 0.05 and passing the parameter test are considered valid. The optimal model for each cycle is determined by the largest stationary R^2^. RMSE: Root Mean Square Error (smaller values indicate better model fit). AIC: Akaike Information Criterion (lower value indicates a better fit).)

**Table 8:** Root Mean Square Error (RMSE) of SARIMA model predictions for the test set of the M_3_ seismic frequency sequence in the Anninghe Fault Zone.

Cycle	Model	Predicted RMSE	MAE	MAPE
Short Cycle	ARIMA(4,1,0) × (0,0,1)_4_	0.7045	0.6420	39.53%
Medium Cycle	ARIMA(2,0,1) × (1,0,0)_20_	0.6868	0.6143	45.45%
Long Cycle	ARIMA(5,1,2) × (1,1,1)_40_	0.7983	0.6941	47.81%

(Note: RMSE = Root Mean Square Error, MAE = mean absolute error, MAPE = mean absolute percentage error (%). Lower values indicate better prediction accuracy. The test set comprises the last 10% of the quarterly sequence (quarters 194–214).)

**Table 9:** Prediction RMSE under different fixed training–test split schemes (training proportions: 90%, 80%, and 70%).

Cycle	Model	Scheme 1 (Original, 90% Training)	Scheme 2 (80% Training)	Scheme 3 (70% Training)	Mean ± SD
Short Cycle	ARIMA(4,1,0) × (0,0,1)_4_	0.7045	0.7189	0.7412	0.7215 ± 0.0154
Medium Cycle	ARIMA(2,0,1) × (1,0,0)_20_	0.6868	0.6957	0.7124	0.6983 ± 0.0106
Long Cycle	ARIMA(5,1,2) × (1,1,1)_40_	0.7983	0.8246	0.8631	0.8287 ± 0.026

**Table 10:** Prediction results of the ARIMA(4,1,0) × (0,0,1)_4_ model for the next 10 steps.

Future Prediction Steps	Predicted Mean	80% Prediction Interval		95% Prediction Interval	
		LCL	UCL	LCL	UCL
1	0.8061	−0.9383	2.5506	−1.8618	3.4741
2	0.6070	−1.1385	2.3525	−2.0625	3.2765
3	0.5808	−1.1649	2.3265	−2.0891	3.2507
4	0.8143	−0.9318	2.5605	−1.8561	3.4848
5	0.8100	−0.9393	2.5592	−1.8653	3.4852
6	0.6179	−1.1326	2.3683	−2.0592	3.2950
7	0.5831	−1.1676	2.3338	−2.0943	3.2606
8	0.7986	−0.9525	2.5497	−1.8794	3.4767
9	0.8120	−0.9419	2.5659	−1.8704	3.4944
10	0.6287	−1.1265	2.3840	−2.0557	3.3131

(Note: LCL = lower confidence limit, UCL = upper confidence limit. Prediction intervals are based on the residual variance of the fitted model. The 80% and 95% intervals indicate the range within which future observations are expected to fall with the given probability.)

**Table 11:** Prediction results of the ARIMA(2,0,1) × (1,0,0)_20_ model for the next 10 steps.

Future Prediction Steps	Predicted Mean	80% Prediction Interval		95% Prediction Interval	
		LCL	UCL	LCL	UCL
1	0.6620	−1.0497	2.3738	−1.9558	3.2799
2	0.6413	−1.0705	2.3531	−1.9766	3.2592
3	0.6660	−1.0458	2.3778	−1.9520	3.2840
4	0.6479	−1.0639	2.3597	−1.9701	3.2659
5	0.6561	−1.0558	2.3679	−1.9620	3.2741
6	0.6503	−1.0615	2.3621	−1.9677	3.2683
7	0.6547	−1.0571	2.3665	−1.9633	3.2727
8	0.6514	−1.0604	2.3633	−1.9666	3.2695
9	0.6622	−1.0496	2.3740	−1.9558	3.2802
10	0.6521	−1.0598	2.3639	−1.9659	3.2701

(Note: LCL = lower confidence limit, UCL = upper confidence limit. Prediction intervals are based on the residual variance of the fitted model. The 80% and 95% intervals indicate the range within which future observations are expected to fall with the given probability.)

**Table 12:** Prediction results of the ARIMA(5,1,2) × (1,1,1)_40_ model for the next 10 steps.

Future Prediction Steps	Predicted Mean	80% Prediction Interval		95% Prediction Interval	
		LCL	UCL	LCL	UCL
1	−0.1871	−2.4040	2.0298	−3.5776	3.2034
2	−0.6270	−2.8481	1.5941	−4.0238	2.7698
3	0.3764	−1.8486	2.6015	−3.0265	3.7793
4	0.5006	−1.7285	2.7296	−2.9085	3.9097
5	−0.6249	−2.8581	1.6082	−4.0402	2.7903
6	1.6283	−0.6088	3.8654	−1.7930	5.0496
7	−0.6256	−2.8667	1.6155	−4.0531	2.8019
8	−0.6247	−2.8698	1.6203	−4.0583	2.8088
9	−0.1888	−2.4379	2.0603	−3.6285	3.2509
10	−0.6250	−2.8781	1.6280	−4.0708	2.8207

(Note: LCL = lower confidence limit, UCL = upper confidence limit. Prediction intervals are based on the residual variance of the fitted model. The 80% and 95% intervals indicate the range within which future observations are expected to fall with the given probability.)

## Data Availability

The seismic catalog data used in this study were obtained from the China Earthquake Networks Center (https://www.cenc.ac.cn/). Detailed data can be queried after registration and login at the National Earthquake Science Data Center database (https://data.earthquake.cn) (Last accessed on 12 March 2026).
